# Presentation of Pediatric Unintentional Injuries at Rural Hospitals in Rwanda: A Retrospective Study

**DOI:** 10.5334/aogh.2711

**Published:** 2020-09-14

**Authors:** Irene Bagahirwa, Madeleine Mukeshimana, Teena Cherian, Theoneste Nkurunziza, Ziad El-Khatib, Jean Claude Byiringiro, Loise Ng’ang’a, Emmanuel Rusingiza, Robert Riviello, Gilles Francois Ndayisaba, Bethany L. Hedt-Gauthier

**Affiliations:** 1 Non-Communicable Diseases Division, Rwanda Biomedical Center, Kigali, RW; 2 University of Rwanda/College of Medicine and Health Sciences, Kigali, RW; 3 Department of Global Health and Social Medicine, Harvard Medical School, Boston, US; 4 Partners In Health/Inshuti Mu Buzima, Kigali, RW; 5 Department of Public Health Sciences, Karolinska Institutet, Solna, SE; 6 World Health Programme, Université du Québec en Abitibi-Témiscamingue (UQAT), Québec, CA; 7 University of Global Health Equity (UGHE), Kigali, RW; 8 Center for Surgery and Public Health, Brigham and Women’s Hospital, Boston, US; 9 University Teaching Hospital of Kigali, Kigali, RW

## Abstract

**Background:**

Injuries are a leading cause of mortality among children globally, with children in low- and middle-income countries more likely to die if injured compared to children in high-income countries. Timely and high-quality care are essential to reduce injury-related morbidity and mortality.

**Objectives:**

This study describes patterns, management, and outcomes of children 0–15 years presenting with unintentional injuries at three district hospitals in rural Rwanda between January 1 and December 31, 2017.

**Methods:**

Using a retrospective cross-sectional study design, we assessed the demographic and clinical characteristics, care provided, and outcomes of the children using data extracted from patient medical charts. We describe the patient population using frequencies and proportions as well as median and interquartile ranges.

**Findings:**

Of the 449 injured children who sought care at the three rural district hospitals, 66.2% (n = 297) were boys. The main causes of injury were falls (n = 261, 58.1%), burns (n = 101, 22.5%), and road traffic injuries (n = 67, 14.9%). Burns were the most common injury among children aged 0–5 years while falls were the leading injury type among the 5–15 years age group. Vital signs were inconsistently completed ranging between 23.8–89.1% of vital sign items. Of the injured children, 37.0% (n = 166) received surgery at the district hospital, general practitioners performed 80.9% (n = 114) of surgeries, 87.4% (n = 145) of operated patients received no anesthesia, and 69.3% (n = 311) were admitted to the district hospital, while 2.7% (n = 12) were transferred to tertiary facilities for higher-level care.

**Conclusions:**

The presentation of child injuries—namely falls, burns, and road traffic accidents—is similar to what has been reported in other sub-Saharan African countries. However, more needs to be done to improve the completion and documentation of vital signs and increase availability of surgical specialists. Finally, targeted strategies to prevent burns and motorcycle-related injuries are recommended prevention interventions for this rural population.

## Background

Injuries are a leading cause of mortality among children, responsible for roughly one million deaths worldwide every year [1]. About 90% of all childhood injuries are unintentional injuries, which include road traffic injuries, falls, burns, drownings, and poisonings (WHO children’s environmental health injuries) [2]. Low- and middle-income countries are disproportionately affected by these injuries, where child mortality due to unintentional injuries is more than three times greater than in high-income countries [3]. With 55 injured children per 100,000 population, the African region bears the highest burden of childhood unintentional injuries in the world [4].

Deaths attributable to injuries increased by more than 50% in East Africa from 1990 to 2010 [5]. Many studies have examined injuries presenting to urban, tertiary hospitals in sub-Saharan Africa [6,7,8,9,10]. In Rwanda’s capital city of Kigali, 22% of all deaths were due to injuries and approximately 60% of these were unintentional injuries [11]. Among pediatric trauma patients presenting to a referral hospital in Kigali, more than half required surgery [10]. While access to surgical services is crucial in the proper management of injuries in rural sub-Saharan Africa, there is still an unmet need of care for surgical conditions, defined as disease states, that require evaluation and management by a surgically-trained provider [12,13]. A study in northern Rwanda found that the population prevalence of untreated surgical conditions was 12%, and about half of these conditions were injuries [14].

Improved understanding of the types of injuries presenting to health facilities is critical for allocating resources needed to treat and manage patients, and for planning injury prevention strategies. Given the paucity of research on pediatric injuries and the subsequent burden on the health sector in rural areas of sub-Saharan Africa, this study aimed to describe the patterns of unintentional injuries, health care services provided, and treatment outcomes among children 0–15 years of age, presenting to three rural district hospitals in Rwanda.

## Methods

## Study setting

This study was conducted in three district hospitals in rural Rwanda: Butaro District Hospital, located in the Northern Province, and Kirehe and Rwinkwavu District Hospitals in the Eastern Province. These hospitals are managed by the Rwanda Ministry of Health (Rwanda-MOH) with technical and financial support from Partners in Health/Inshuti Mu Buzima (PIH/IMB), a nongovernmental organization that has been supporting the Rwanda-MOH since 2005. Together, the three hospitals serve a population of approximately 800,000 people.

Rwanda’s health care system is tiered, such that individuals requiring medical attention, including injured children, first seek care from the nearest health center. Health centers provide basic services including wound care and pain medication. For conditions that need advanced management, patients are referred to the nearest district hospital. These district hospitals are staffed with a higher cadre of health care providers, including nurses with more advanced training and general practitioners. If further specialized care is needed, the patient is referred from the district hospital to the respective national referral hospitals. Close to three-quarters of Rwandans have health insurance, of which 97% are covered by community-based health insurance (CBHI), a public insurance that pays for 90% of all medical expenses at public facilities [15].

## Study design and population

This cross-sectional retrospective study included all children ages of 0–15 years who presented with unintentional injuries at Butaro, Kirehe, and Rwinkwavu District Hospitals between January 1 and December 31, 2017. For this study, we referred to the WHO definition of unintentional injury including road traffic injuries, drownings, burns, falls, poisonings, and animal-related injuries [1].

## Data collection and analysis

We extracted demographic data, clinical characteristics, details on care provided, and outcomes (including hospitalization, discharge, and referral) from patients’ medical charts. We recorded data directly into Ona^®^, a mobile data collection application [16]. Patient files from the three district hospitals undergo regular quality audits by data managers as part of the process of monthly reporting to the Rwanda-MOH. Additionally, to ensure the highest data quality possible, we trained data collectors on extracting and entering data from medical charts to the Ona^®^ database, which contained embedded validation criteria and ranges.

We described categorical variables using frequencies and proportions and continuous variables using medians and interquartile ranges (IQR). Children’s ages were categorized as less than 5 years, 5–10 years, and 11–15 years. We conducted data analyses using Stata^®^ version 15 (StataCorp, College Station, Texas, USA).

### Ethics

We received scientific approval from the PIH/IMB Research Committee, and ethical approval from the Rwanda National Ethic Committee (Kigali, Rwanda; No. 102/RNEC/2018:) and Partners Institutional Review Board (Boston, USA, No. 2013P000047).

## Results

Our study included 449 children. For the 447 with age recorded, the children were approximately evenly distributed by age groups: 142 (31.8%) aged 0–5 years, 171 (38.3%) aged 5–10 years, and 134 (30.0%) aged 11–15 years (Table 1). Two-thirds (n = 297, 66.1%) were boys. Kirehe District Hospital had the highest burden of injured children, representing 292 (65.0%) of the cases reported. Nearly all children (426, 94.9%) had some type of insurance, primarily the public insurance CBHI.

**Table 1:** Demographic characteristics of children (N = 449).

Characteristic	n	%

Age (n = 447)		
<5 years	142	31.8
5–10 years	171	38.2
11–15 years	134	30.0
Gender		
Boys	297	66.1
Girls	152	33.9
District Hospital		
Kirehe	292	65.0
Rwinkwavu	96	21.4
Butaro	61	13.6
Type of insurance		
CBHI* (Mutuelle)	348	77.5
Private insurance	78	17.4
No insurance	23	5.1

* CBHI: Community Based Health Insurance.

The majority of the injuries presented to the district hospitals were falls (n = 261, 58.1%), followed by burns (n = 101, 22.5%), road traffic accidents (n = 67, 14.9%), and animal-related injuries (n = 20, 4.5%) (Table 2). The type of injury varied by age, with burns being the most common injury in children under five (n = 66 out of 142, 46.5%), followed by falls (n = 57; 40.1%) (results not shown). Falls were the most common injury type for children aged 5–10 years (n = 106 out of 171, 62%) and aged 11–15 years (n = 98 out of 134, 73.1%).

**Table 2:** Cause and presentation of injuries for children in rural Rwandan district hospitals (N = 449).

	n	%

**Description of injuries**		
Injury type		
Fall	261	58.1
Burn	101	22.5
Road traffic accident	67	14.9
Animal-related injury	20	4.5
Cause of burn (n = 100)		
Hot liquids	89	89.0
Fire burns	8	8.0
Electrical	2	2.0
Insecticides	1	1.0
Vehicle involved in road traffic accident (n = 67)		
Motorcycle	56	83.6
Car	11	16.4
Child involved in accident (n = 52)		
Pedestrian	38	73.1
Passenger	14	26.9
Cause of animal-related injury (n = 20)		
Dog bite	11	55.0
Snake bite	7	35.0
Hit/bitten by domestic animal (not dog)	1	5.0
Hit/bitten by wild animal (not dog)	1	5.0
**Presentation of injuries**		
Patient’s status on arrival (n = 432)		
Stable†	383	88.7
Confused	45	10.4
Coma	4	0.9
Patient’s primary diagnosis of injury		
Closed fracture/dislocation	172	38.3
Open fracture	117	26.1
Burn	92	20.5
Soft tissue injury	30	6.7
Head/spine injury	25	5.6
Infection	3	0.7
Abdominal and thoracic injury	2	0.4
Other	8	1.8

† Stable refers to the patient status in which his/her consciousness is lucid. Not in coma nor confused.

Hot liquids were the most common cause of burn injuries (n = 89, 89.0%) (Table 2). Road accidents were most commonly caused by motorcycles (n = 56, 83.6%) and most affected pedestrians (n = 38 out of 52, 73.1%). Dog bites were the most common type of animal-related injuries (n = 11, 55.0%) followed by snake bites (n = 7, 35.0%). The majority of the injured children (n = 383, 88.7%) were stable when presenting to the district hospital. More than half had a fracture or dislocation, with 117 (26.1%) having an open fracture and 172 (38.3%) having a closed fracture or dislocation.

Table 3 depicts the clinical care received by the children at the district hospitals. The following vital signs were measured and recorded in injured children’s charts: heart rate (n = 400, 89.1%), blood pressure (n = 107, 23.8%), breathing rate (n = 354, 78.8%), and Glasgow Coma Scale score (n = 214, 47.7%). Only 142 children (31.6%) received an X-ray, and no other imaging options were available at the district hospitals. Approximately one-third of children (n = 166, 37.0%) received surgical procedures; these included closed reduction and immobilization (n = 120, 72.3%), wound debridement and suture (n = 36, 21.7%), and exploratory laparotomy (n = 10; 6.0%). General practitioners were the main cadre of health care providers who performed surgical services (n = 114, 80.9%).

**Table 3:** Clinical care provided to injured children at the district hospital (N = 449).

Type of care provided	n	%

**Vital signs and exams**		
Heart rate recorded	400	89.1
Blood pressure recorded	107	23.8
Breath rate recorded	354	78.8
Glasgow Coma Scale score recorded	214	47.7
Imagery exam done	142	31.6
Blood tests performed	49	10.9
**Non-surgical care**		
Intra Venous fluid administered	70	15.6
Blood transfusion conducted	9	2.0
Antibiotics administered	210	46.8
Pain medication administered	388	86.4
Wound dressed	250	55.7
Anti-venom for patients with snake bite (n = 7)	7	100
Anti-rabies for patients with animal bite (n = 13)	9	69.2
**Surgical care**		
Patients who received surgery	166	37.0
Type of surgical service (n = 166)		
Closed reduction and immobilization	120	72.3
Wound debridement and suture	41	24.7
Exploratory laparotomy	10	6.0
Other surgery	6	3.6
Surgical provider’s cadre (n = 141)		
General Practitioner	114	80.9
Specialist	6	4.3
More than one cadre	21	14.9
Type of anesthesia used (n = 166)		
No anesthesia	145	87.4
Loco-regional anesthesia	15	9.0
General anesthesia	6	3.6
ASA class* for patients who received anesthesia (n = 21)	21	100
Surgical complications	3	1.8
Type of surgical complication (n = 3)		
Surgical site infection	1	33.3
Poor bone alignment	1	33.3
Other surgical complication	1	33.3

* American Society of Anesthesiologists Classification.

The majority of child injury cases were admitted to the district hospitals (n = 311, 69.3%) (Table 4). The median length of stay at the hospital was five days (IQR = 3–10, n = 297). Only 12 children (2.7%) were referred directly to tertiary level, for reasons that included lack of specialists (n = 5 out of 7, 71.4%) and lack of indicated medication (n = 4 out of 7, 57.1%).

**Table 4:** Outcomes among injured children (N = 449).

Outcome	n	%

Patient outcomes		
Successfully managed at outpatient clinic and discharged	115	25.6
Admitted to district hospital	311	69.3
Admitted to district hospital while waiting for appointment at referral hospital	10	2.2
Referred directly to tertiary care	12	2.7
Absconded from care	1	0.2
Length of stay at district hospital, in days; median (IQR) (n = 297)	5 (3–10)	
Reason for referral to tertiary hospital (n = 7)*		
Lack of specialists	5	71.4
Lack of drugs	4	57.1
Family preference	1	14.3
Better management	1	14.3

* Percentages exceed 100% because one patient could have been referred due to more than one reason.

## Discussion

This study characterized the most common unintentional injuries in children presenting to three district hospitals in rural Rwanda. Consistent with other studies, burns were the most common type of unintentional injury among children aged 0–5 years [17,18,19,20,21,22]. This can be attributed to their exposures during cooking by their mothers and caregivers, given that portable stove and open fires are the main means for cooking in Rwanda and many other sub-Saharan African countries [20,23,24,25]. Burn prevention strategies. such as culturally sensitive home visitation and education programs by community health workers, and educational interventions at schools, should be explored in Rwanda to reduce the burden of these injuries on children. Future studies should explore the types and management of burns at the district hospitals [26,27,28].

Road traffic accidents constituted 15% of all child injuries and was the most common unintentional injury in children over five years. Road traffic accidents are a common cause of injury for both children and adults in sub-Saharan Africa [29]. Similar to other studies in the region, we found that most injured children were pedestrians [30,31,32]. We suspect that children are vulnerable to traffic accidents while playing or going to and returning from school, a hypothesis supported by a study in Tanzania [29]. Kirehe District, which had the highest burden of injuries, is located at the border of Rwanda and Tanzania, experiencing increased traffic of different types of motor vehicles, increasing the risk of injury to pedestrians. Rwanda has implemented efforts to prevent road traffic injuries, including restricting public transport to officially registered vehicles, installing governor devices to limit the speed of vehicles, and strictly enforcing laws requiring helmets when riding motorcycles [33]. We recommend additional studies to explore the reasons for high pedestrian injuries in school-aged populations, and to identify viable interventions to reduce pedestrian-related road traffic accidents.

Most of the injured children were treated and admitted to the district hospital, and only a few were referred to tertiary facilities for care. Low referral rates reduce the financial and time burden on the families to get care at a tertiary facility. These facilities are primarily located in Kigali, 2–3 hour’s drive from the included district hospitals. We found that the care documentation practices at the district hospitals were not always optimal, particularly for vital signs, which mirrors findings from another study in Rwanda [9]. Lack of documentation indicates that vital signs are not being collected or are not being recorded. This hinders longitudinal management of patients and can compromise patient care and outcomes. Systems such as the Modified Early Warning Score (MEWS) or modified version of the WHO checklist for trauma care, better training for nurses, and regular quality control are strategies for improving the collecting and recording of vital signs [34,35,36,37,38].

There were further limitations of infrastructure—for example, the only imaging available at the hospitals was X-rays, and was only conducted on 31.6% of all children and 39.1% of those with fractures. This limited provision of imaging may reflect both inadequate infrastructure and trained personnel to administer imaging, as observed in other settings [13]. Other imaging devices, such as Computed Tomography (CT) and Magnetic Resonance Imaging (MRI), are common for management of injuries in high-income settings. The costs and training required for such devices is prohibitive for use in rural African district hospitals, and is not currently on the list of recommended technology for district hospitals in Rwanda. However, more research into viable technologies for this setting and greater investment in reliable availability of simple X-ray technology could facilitate the district hospitals’ abilities to optimally care for these children. In addition, studies on risk factors, management, and outcome of fracture patients are recommended.

General practitioners performed the majority of surgeries for injured patients, reflecting a gap in availability of surgical specialists in Rwanda. In addition, only one-fifth of the patients received anesthesia, and anesthetic complications were not reported. Rwanda has recognized this gap of health personnel and introduced medical and nursing specialties training programs with support from Human Resources for Health [39]. These surgical specialties programs include Masters in Surgical Needs, Masters in Medical Surgical Nursing, and Masters in Perioperative Nursing [39]. More training of more specialists in surgical care is recommended.

## Limitations

A limitation of the study is using routinely collected data from patients’ medical charts, which may have incomplete data, or may not include other variables of interest (for example we did not have data on patients’ economic status). However, our results derived from data available at these hospitals give a glimpse into the current pediatric injury management practices at rural district hospitals in Rwanda, and demonstrate avenues to improve care provision. A second limitation is that data were collected on injured children who presented at district hospitals for care, meaning we did not capture those with minor injuries who presented to health centers, those who had severe injuries and sought care directly from tertiary level hospitals, those who never sought care or those who died before presenting to a health facility. Although this means our findings may underestimate the number of unintentionally injured children, the results further underscore the need to improve care related to pediatric injuries in rural Rwanda. A third limitation is that this study only looked at the outcomes that were documented on patient charts, and therefore does not provide a comprehensive outcome of injuries. For example, we did not monitor outcomes following a patient’s discharge from the district hospital or referral to tertiary facilities. In addition, we did not collect information on long-term disability. Future studies should include prospective data that follows up with injured children to obtain additional details on outcomes, as well as assess the risk factors for longer duration of stay in the hospital.

## Conclusions

Pediatric unintentional injuries constitute a great threat to children in rural Rwanda. We found that children are affected by preventable injuries such as falls, road traffic accidents and burns. Although most injured children were treated at district hospitals, we found absence and/or poor recording of vital signs, which may hinder injury management. Future studies should explore steps to improve documentation, including regular data quality audit and nurse trainings at the district hospital level. Finally, continuing to strengthen existing prevention strategies and implement new ones to promote safety against fires at home and motorcycle collisions on the road represent high-yield opportunities for improved outcomes for children.

## Data Accessibility Statements

The datasets used and/or analyzed during the current study are available from the corresponding author on reasonable request.
